# Disproportionation of elemental sulfur by *Exiguobacterium* from marine sediment

**DOI:** 10.1093/ismeco/ycaf168

**Published:** 2025-09-24

**Authors:** Xiao-Tong Wu, Min Qiu, Yu-Qin He, Kun Wu, Jun-Yi Zhao, Juan Wang, Hong-Yun Ren, Jing-Yun Su, Peng Bao

**Affiliations:** Key Laboratory of Urban Environment and Health, Ningbo Observation and Research Station, Institute of Urban Environment, Chinese Academy of Sciences, Xiamen 361021, P. R. China; University of Chinese Academy of Sciences, Beijing 100049, P. R. China; Key Laboratory of Urban Environment and Health, Ningbo Observation and Research Station, Institute of Urban Environment, Chinese Academy of Sciences, Xiamen 361021, P. R. China; University of Chinese Academy of Sciences, Beijing 100049, P. R. China; College of Life Sciences, Fujian Agriculture and Forestry University, 350002 Fuzhou, P. R. China; Key Laboratory of Urban Environment and Health, Ningbo Observation and Research Station, Institute of Urban Environment, Chinese Academy of Sciences, Xiamen 361021, P. R. China; University of Chinese Academy of Sciences, Beijing 100049, P. R. China; Key Laboratory of Urban Environment and Health, Ningbo Observation and Research Station, Institute of Urban Environment, Chinese Academy of Sciences, Xiamen 361021, P. R. China; University of Chinese Academy of Sciences, Beijing 100049, P. R. China; Key Laboratory of Urban Environment and Health, Ningbo Observation and Research Station, Institute of Urban Environment, Chinese Academy of Sciences, Xiamen 361021, P. R. China; University of Chinese Academy of Sciences, Beijing 100049, P. R. China; Key Laboratory of Urban Environment and Health, Ningbo Observation and Research Station, Institute of Urban Environment, Chinese Academy of Sciences, Xiamen 361021, P. R. China; University of Chinese Academy of Sciences, Beijing 100049, P. R. China; Key Laboratory of Urban Environment and Health, Ningbo Observation and Research Station, Institute of Urban Environment, Chinese Academy of Sciences, Xiamen 361021, P. R. China; University of Chinese Academy of Sciences, Beijing 100049, P. R. China; Key Laboratory of Urban Environment and Health, Ningbo Observation and Research Station, Institute of Urban Environment, Chinese Academy of Sciences, Xiamen 361021, P. R. China; University of Chinese Academy of Sciences, Beijing 100049, P. R. China; College of Life Sciences, Fujian Agriculture and Forestry University, 350002 Fuzhou, P. R. China; Key Laboratory of Urban Environment and Health, Ningbo Observation and Research Station, Institute of Urban Environment, Chinese Academy of Sciences, Xiamen 361021, P. R. China; University of Chinese Academy of Sciences, Beijing 100049, P. R. China

**Keywords:** sulfur disproportionation, bacilli, rhodanese, elemental biogeochemical cycle

## Abstract

Elemental sulfur disproportionation is an ancient microbial metabolic process, and the phylogenetic distribution of elemental sulfur disproportionators may be broader than previously thought. We enriched a bacterial community capable of this process, with *Exiguobacterium* making up 99.45% of the total population. The results indicate that *Exiguobacterium* facilitates the formation of thiosulfate and sulfide through elemental sulfur disproportionation. This study represents the first report documenting elemental sulfur disproportionation by Bacilli. Metagenomic analysis shows that rhodanese-like sulfur transferase genes are significantly more abundant in the experimental group than in the control group, suggesting that they are implicated in elemental sulfur disproportionation in *Exiguobacterium.* These findings support the idea that Bacilli and/or Firmicutes are the oldest extant bacterial phyla. Our research fills a critical gap in understanding sulfur biogeochemical cycles. Given the widespread occurrence of *Exiguobacterium* across various environments, direct microbial transformations between elemental sulfur and thiosulfate are likely prevalent throughout ecological systems.

## Introduction

Sulfur-disproportionating microorganisms are essential to the biogeochemical sulfur cycle, facilitating the disproportionation of inorganic sulfur compounds. These microorganisms include members of the Clostridia class within the Bacillota phylum (formerly known as Firmicutes), as well as representatives from the Thermodesulfobacteriota order, which comprises both original Thermodesulfobacteria and various taxa from the delta-Proteobacteria group [1, 2]. To date, very few sulfur-disproportionating bacteria have been classified within gamma-Proteobacteria and Nitrospirota [1, 3]. These microorganisms inhabit a variety of environments, including freshwater ecosystems [4], shallow marine sediments [5], hypersaline lakes and alkaline lakes [6], along with hydrothermal ecosystems [7].

Different types of sulfur-disproportionating microorganisms employ distinct functional enzymes during various sulfurous species (e.g. elemental sulfur, sulfite, and thiosulfate) disproportionation processes [1]. Rhodanese-like sulfur transferases, thiosulfate reductases, and polysulfide reductases (phsA/psrA) are essential in facilitating the disproportionation of elemental sulfur [1]. Importantly, elemental sulfur disproportionation is recognized as an ancient microbial metabolism that predates even sulfate reduction [8]. The phylogenetic distribution of elemental sulfur disproportionators may be more extensive than previously understood. The discovery of elemental sulfur disproportionators will contribute to our understanding of primordial microbial and life origin processes, as well as sulfur biogeochemical cycles on both primordial and modern Earth.

## Results and discussion

**Figure 1:**
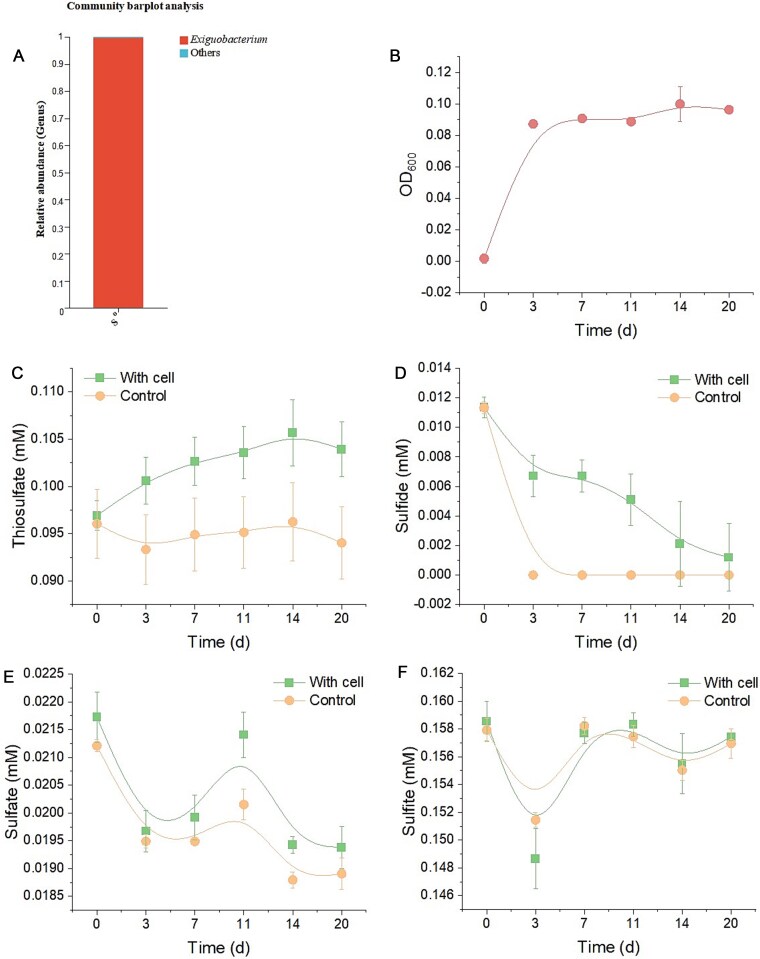
The bacterial community structure showed that *Exiguobacterium* occupied the main component (A). The bacterial growth curve based on OD_600_ (B). Sulfur transformation in bacterial and control groups (C–F). The error bars represent standard errors calculated from three replicates.

Microbial diversity analysis reveals that *Exiguobacterium* constitutes 99.45% of the total bacterial community (Fig. 1A). Furthermore, the structure of the bacterial community exhibited stability throughout the growth period. Figure 1B illustrates a subtle yet significant increase in bacterial cell numbers during elemental sulfur disproportionation. The production of thiosulfate and dissolved sulfide during this process is depicted in Fig. 1C and D. Notably, we detected dissolved sulfide within the bacterial group at the onset of cultivation (Fig. 1D), which can be attributed to the addition of 0.1 mM sulfide to eliminate residual oxygen and initiate the disproportionation reaction. A comparison with the control group indicates continuous sulfide production in the bacterial community (Fig. 1D). The absence of accumulated dissolved sulfide may result from its cleavage from S^0^-ring structures derived from elemental sulfur via nucleophilic attack, leading to polysulfides (Equation 1). It is plausible that more sulfide exists within the system than our detection methods can reveal. Concurrently, we monitored background sulfate and sulfite concentrations and observed no significant differences between both groups (Fig. 1E and F). These results suggest that elemental sulfur disproportionation within the bacterial community adheres to reaction equation 2 [9], facilitated by *Exiguobacterium*. To our knowledge, elemental sulfur disproportionation typically yields sulfate and sulfide; thus, this study represents a novel contribution regarding thiosulfate and sulfide formation through such processes. Elemental sulfur and thiosulfate are critical intermediates with rapid biological turnover in global sulfur cycling [10, 11]; however, direct microbial transformation between these two compounds remains sparsely documented. Our findings address this gap in understanding microbial sulfur cycling.


(1) \begin{align*}\kern-3pc {\mathrm{S}}_8^0+{\mathrm{H}\mathrm{S}}^{-}\rightleftarrows{\mathrm{S}}_8{\mathrm{S}}^{2-}+{\mathrm{H}}^{+} \end{align*}

(2) \begin{equation*} {\displaystyle \begin{array}{c}4{\mathrm{S}}^0+4{\mathrm{O}\mathrm{H}}^{-}\to{\mathrm{S}}_2{\mathrm{O}}_3^{2-}+2{\mathrm{H}\mathrm{S}}^{-}+{\mathrm{H}}_2\mathrm{O}\end{array}} \end{equation*}

ΔG^0^ (kJ/mol) = −119.91

The versatile genus *Exiguobacterium* comprises a diverse group of pigmented, gram-positive bacteria exhibiting variable morphologies. Members of the *Exiguobacterium* genus are highly varied and inhabit a multitude of environments, including soils, sediments, seawater, glaciers, and hydrothermal vents [12]. Given the extensive distribution of *Exiguobacterium*, these organisms play a crucial role in the biogeochemical cycling of sulfur.

**Figure 2:**
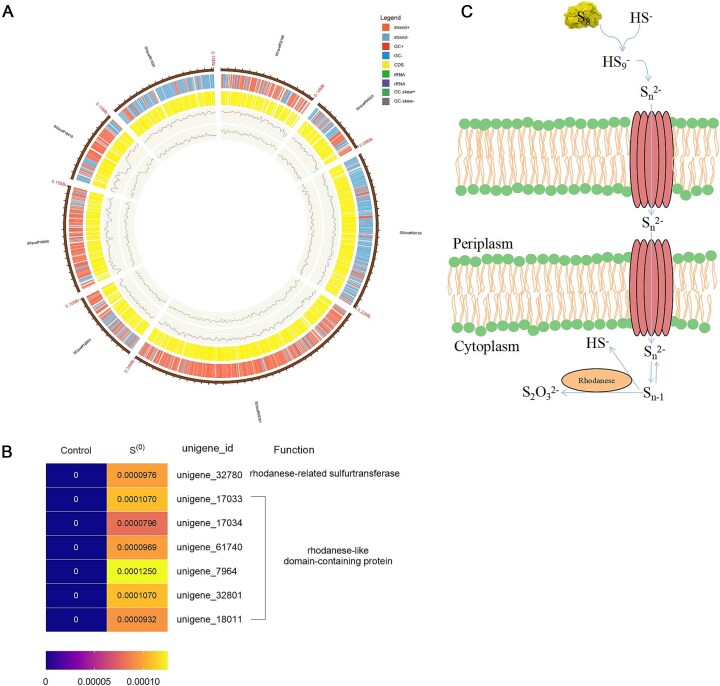
Bin-based genetic map of *Exiguobacterium* (A). The outermost number was the sequence length in bin, followed by the gene locations on the positive chain, the gene locations on the negative chain, coding sequence (CDS) locations, and tRNA locations. Metagenomic analysis of targeting functional genes associated with elemental sulfur disproportionation in *Exiguobacterium* cultivated in basal medium supplemented with ether sulfite (initial bacterial enrichment), or elemental sulfur (B). Summary scheme of the possible strategy adopted by *Exiguobacterium* to uptake and disproportionate elemental sulfur (C).

The genome size of *Exiguobacterium* was assembled to be 2.65 Mb (Fig. 2A), comprising 2686 genes with an average gene length of 869 bp. Metagenomic analysis targeting functional genes associated with elemental sulfur disproportionation reveals that rhodanese-related sulfurtransferase genes and rhodanese-like domain-containing protein-coding genes are clearly contained in *Exiguobacterium* genome (Fig. 2B). Thiosulfate reductases, and polysulfide reductases (phsA/psrA) were not found in *Exiguobacterium* genome.

The structural modules of rhodanese are widely distributed across the three primary evolutionary phyla. A substantial body of evidence suggests that these modules function as versatile carriers of sulfur, adapting their roles to fulfill the demand for reactive sulfane sulfur in various metabolic and regulatory pathways [13]. The rhodanese enzyme is likely to have been present in the last common ancestor of bacteria and archaea, from which it has been vertically inherited by both domains [14]. Rhodanese-like sulfur transferases are believed to play a significant role in elemental sulfur disproportionation [1, 15]. Polysulfides may serve as intermediates facilitating the uptake of elemental sulfur into the cytoplasm while also functioning as sulfide scavengers (Fig. 2C) [16]. In principle, elemental sulfur can spontaneously form from polysulfides within the cytoplasm where rhodanese-like sulfur transferases reside and be disproportionated by them (Equation 3) (Fig. 2C) [17]. To date, no research has documented the sulfur disproportionation capabilities of *Exiguobacterium* or its members within the Bacilli class. This finding supports the hypothesis that Bacilli and/or Firmicutes represent some of the oldest extant bacterial phyla [18, 19].


(3) \begin{equation*} {\displaystyle \begin{array}{c}{\mathrm{S}}_{\mathrm{n}}^{2-}+{\mathrm{H}}^{+}\rightleftarrows{\mathrm{H}\mathrm{S}}^{-}+{\mathrm{S}}_{\mathrm{n}-1}\end{array}} \end{equation*}

In this study, we enriched a bacterial community capable of elemental sulfur disproportionation, in which *Exiguobacterium* comprises 99.45% of the total bacterial population. The results indicate that the formation of thiosulfate and sulfide through elemental sulfur disproportionation is mediated by *Exiguobacterium*. This research represents the first report documenting the generation of thiosulfate and sulfide via bacterial elemental sulfur disproportionation, thereby bridging a significant gap in our comprehension. Given the widespread distribution of *Exiguobacterium* across various environments, direct microbial transformation between elemental sulfur and thiosulfate is likely to be prevalent within ecosystems. Metagenomic analysis suggests that rhodanese-like sulfur transferase genes are implicated in elemental sulfur disproportionation in *Exiguobacterium.* These findings further support the hypothesis that the phyla Bacillus and/or Firmicutes are the oldest extant bacterial phyla.

## Data Availability

The data is publicly available at the NCBI database. Metagenome-assembled genome of *Exiguobacterium* are under the Bioproject PRJNA1159524 (BioSample accessions: SRX26052527). Unassembled metagenome reads are under the Bioproject PRJNA1309488 (BioSample accessions: SRR35156687). The 16S reads are under the Bioproject PRJNA1309172 (BioSample accessions: SRA: SRR35082637).
